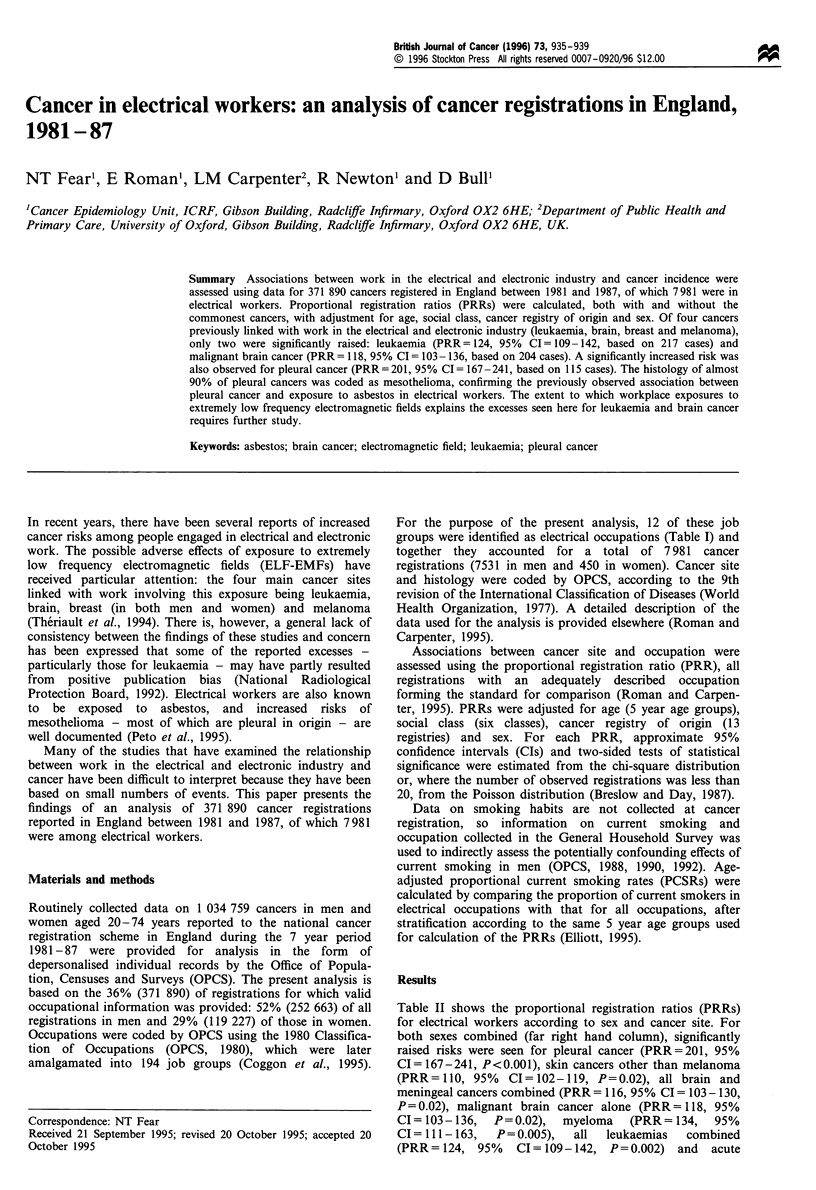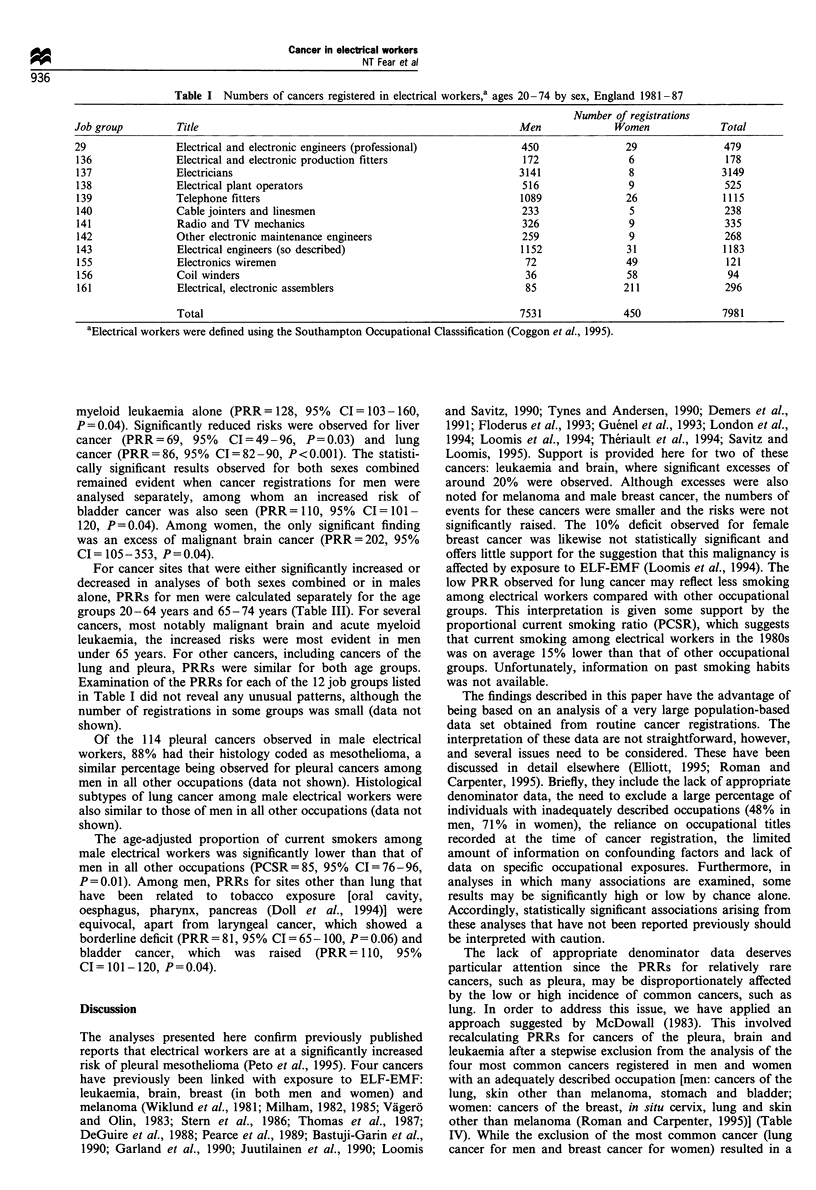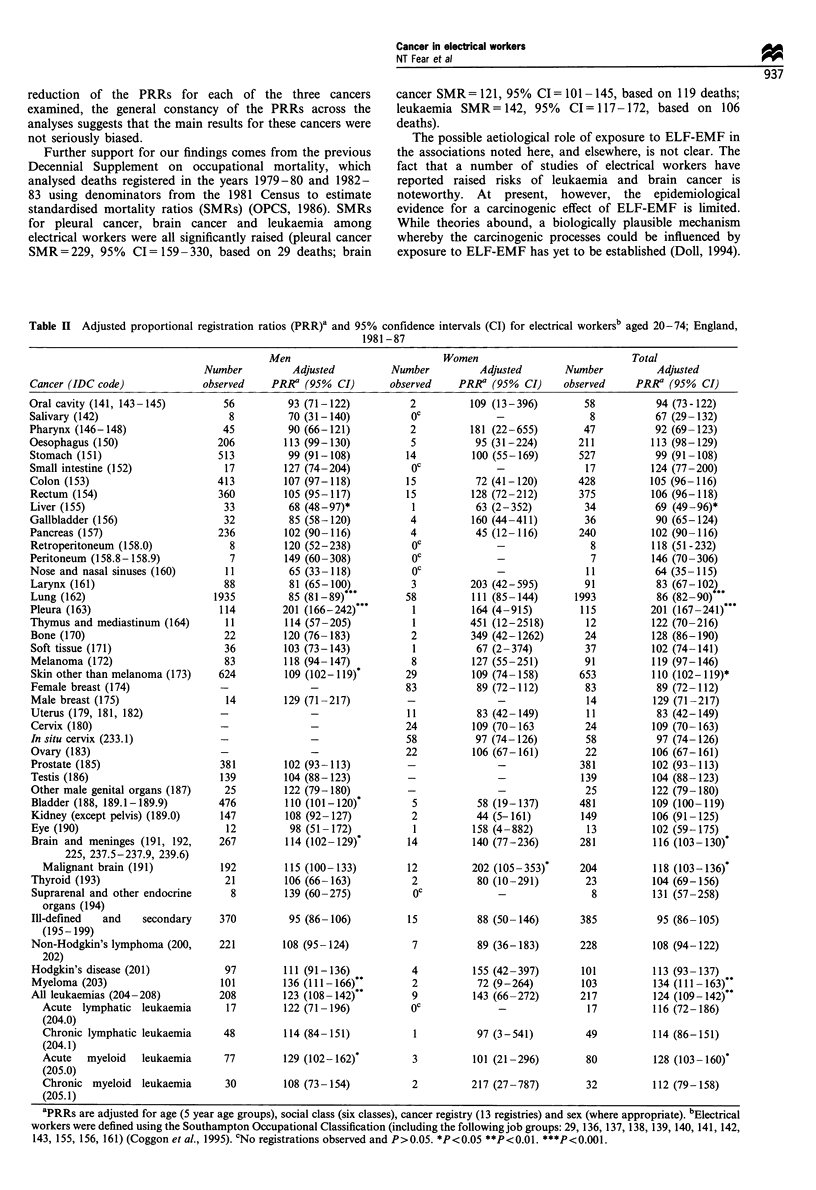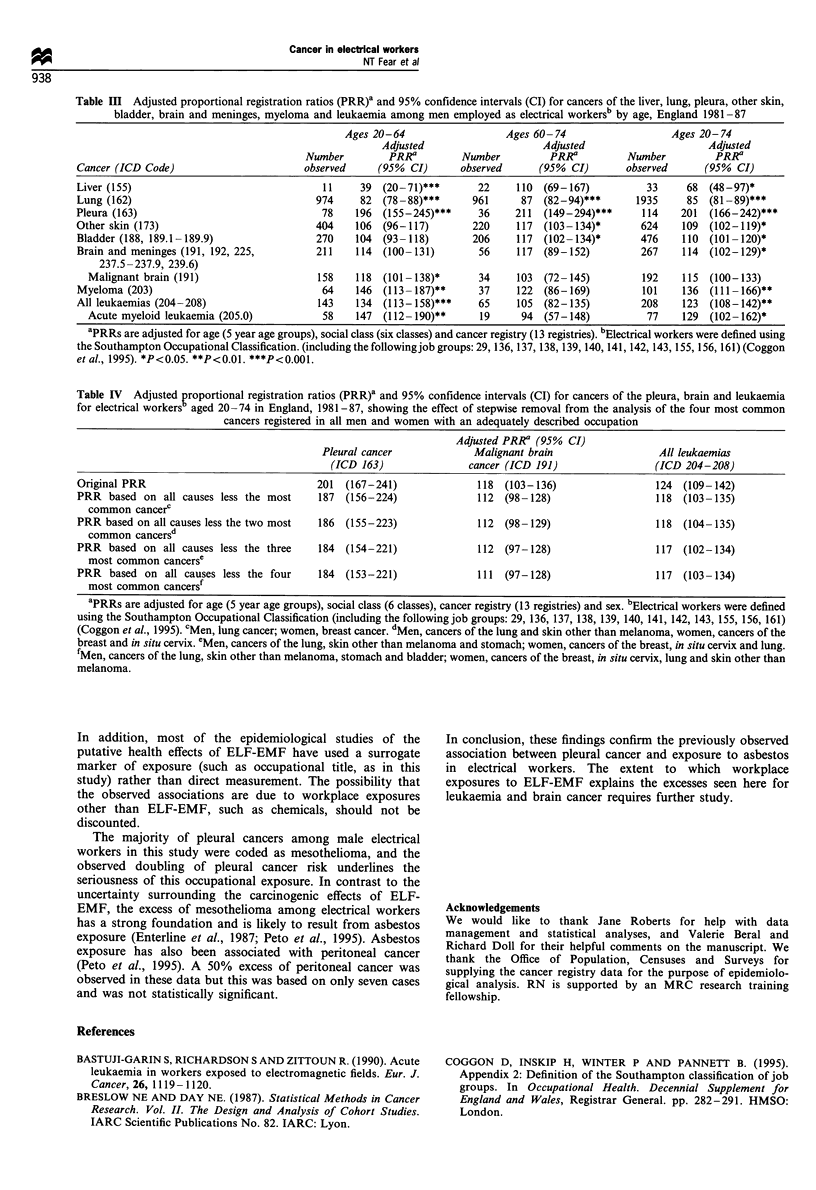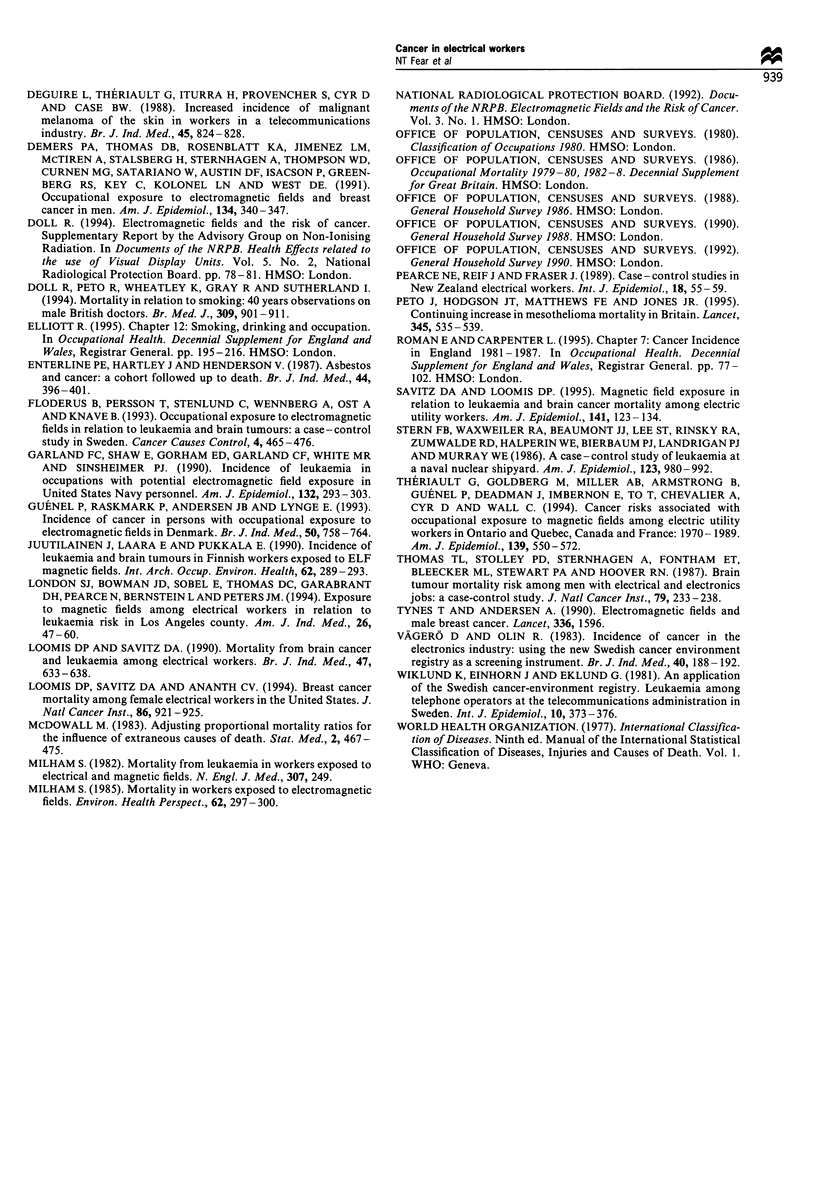# Cancer in electrical workers: an analysis of cancer registrations in England, 1981-87.

**DOI:** 10.1038/bjc.1996.167

**Published:** 1996-04

**Authors:** N. T. Fear, E. Roman, L. M. Carpenter, R. Newton, D. Bull

**Affiliations:** Cancer Epidemiology Unit, ICRF, Radcliffe Infirmary, Oxford, UK.

## Abstract

Associations between work in the electrical and electronic industry and cancer incidence were assessed using data for 371 890 cancers registered in England between 1981 and 1987, of which 7981 were in electrical workers. Proportional registration ratios (PRRs) were calculated, both with and without the commonest cancers, with adjustment for age, social class, cancer registry of origin and sex. Of four cancers previously linked with work in the electrical and electronic industry (leukaemia, brain, breast and melanoma), only two were significantly raised: leukaemia (PRR=124, 95% CI=109-142, based on 217 cases) and malignant brain cancer (PRR=118, 95% CI=103-136, based on 204 cases). A significantly increased risk was also observed for pleural cancer (PRR=201, 95% CI=167-241, based on 115 cases). The histology of almost 90% of pleural cancers was coded as mesothelioma, confirming the previously observed association between pleural cancer and exposure to asbestos in electrical workers. The extent to which workplace exposures to extremely low frequency electromagnetic fields explains the excesses seen here for leukaemia and brain cancer requires further study.


British Journal of Cancer (1996) 73, 935-939
© 1996 Stockton Press All rights reserved 0007-0920/96 $12.00

### Cancer in electrical workers: an analysis of cancer registrations in England, 1981-87

NT Fear[1], E Roman[1], LM Carpenter[2], R Newton[1] and D Bull[1]

[1]*Cancer Epidemiology Unit, ICRF, Gibson Building, Radcliffe Infirmary, Oxford OX2 6HE;* [2]*Department of Public Health and Primary Care, University of Oxford, Gibson Building, Radcliffe Infirmary, Oxford OX2 6HE, UK.*

**Summary** Associations between work in the electrical and electronic industry and cancer incidence were assessed using data for 371 890 cancers registered in England between 1981 and 1987, of which 7 981 were in electrical workers. Proportional registration ratios (PRRs) were calculated, both with and without the commonest cancers, with adjustment for age, social class, cancer registry of origin and sex. Of four cancers previously linked with work in the electrical and electronic industry (leukaemia, brain, breast and melanoma), only two were significantly raised: leukaemia (PRR = 124, 95% CI = 109-142, based on 217 cases) and malignant brain cancer (PRR = 118, 95% CI = 103-136, based on 204 cases). A significantly increased risk was also observed for pleural cancer (PRR = 201, 95% CI = 167-241, based on 115 cases). The histology of almost 90% of pleural cancers was coded as mesothelioma, confirming the previously observed association between pleural cancer and exposure to asbestos in electrical workers. The extent to which workplace exposures to extremely low frequency electromagnetic fields explains the excesses seen here for leukaemia and brain cancer requires further study.

**Keywords:** asbestos; brain cancer; electromagnetic field; leukaemia; pleural cancer

In recent years, there have been several reports of increased cancer risks among people engaged in electrical and electronic work. The possible adverse effects of exposure to extremely low frequency electromagnetic fields (ELF-EMFs) have received particular attention: the four main cancer sites linked with work involving this exposure being leukaemia, brain, breast (in both men and women) and melanoma (Thériault *et al.*, 1994). There is, however, a general lack of consistency between the findings of these studies and concern has been expressed that some of the reported excesses - particularly those for leukaemia - may have partly resulted from positive publication bias (National Radiological Protection Board, 1992). Electrical workers are also known to be exposed to asbestos, and increased risks of mesothelioma - most of which are pleural in origin - are well documented (Peto *et al.*, 1995).

Many of the studies that have examined the relationship between work in the electrical and electronic industry and cancer have been difficult to interpret because they have been based on small numbers of events. This paper presents the findings of an analysis of 371 890 cancer registrations reported in England between 1981 and 1987, of which 7 981 were among electrical workers.

### Materials and methods

Routinely collected data on 1 034 759 cancers in men and women aged 20-74 years reported to the national cancer registration scheme in England during the 7 year period 1981-87 were provided for analysis in the form of depersonalised individual records by the Office of Population, Censuses and Surveys (OPCS). The present analysis is based on the 36% (371 890) of registrations for which valid occupational information was provided: 52% (252 663) of all registrations in men and 29% (119 227) of those in women. Occupations were coded by OPCS using the 1980 Classification of Occupations (OPCS, 1980), which were later amalgamated into 194 job groups (Coggon *et al.*, 1995). For the purpose of the present analysis, 12 of these job groups were identified as electrical occupations (Table I) and together they accounted for a total of 7 981 cancer registrations (7531 in men and 450 in women). Cancer site and histology were coded by OPCS, according to the 9th revision of the International Classification of Diseases (World Health Organization, 1977). A detailed description of the data used for the analysis is provided elsewhere (Roman and Carpenter, 1995).

Associations between cancer site and occupation were assessed using the proportional registration ratio (PRR), all registrations with an adequately described occupation forming the standard for comparison (Roman and Carpenter, 1995). PRRs were adjusted for age (5 year age groups), social class (six classes), cancer registry of origin (13 registries) and sex. For each PRR, approximate 95% confidence intervals (CIs) and two-sided tests of statistical significance were estimated from the chi-square distribution or, where the number of observed registrations was less than 20, from the Poisson distribution (Breslow and Day, 1987).

Data on smoking habits are not collected at cancer registration, so information on current smoking and occupation collected in the General Household Survey was used to indirectly assess the potentially confounding effects of current smoking in men (OPCS, 1988, 1990, 1992). Age-adjusted proportional current smoking rates (PCSRs) were calculated by comparing the proportion of current smokers in electrical occupations with that for all occupations, after stratification according to the same 5 year age groups used for calculation of the PRRs (Elliott, 1995).

### Results

Table II shows the proportional registration ratios (PRRs) for electrical workers according to sex and cancer site. For both sexes combined (far right hand column), significantly raised risks were seen for pleural cancer (PRR = 201, 95% CI = 167-241, $P<0.001$), skin cancers other than melanoma (PRR = 110, 95% CI = 102-119, $P=0.02$), all brain and meningeal cancers combined (PRR = 116, 95% CI = 103-130, $P=0.02$), malignant brain cancer alone (PRR = 118, 95% CI = 103-136, $P=0.02$), myeloma (PRR = 134, 95% CI = 111-163, $P=0.005$), all leukaemias combined (PRR = 124, 95% CI = 109-142, $P=0.002$) and acute

Correspondence: NT Fear
Received 21 September 1995; revised 20 October 1995; accepted 20 October 1995

**Table I** Numbers of cancers registered in electrical workers,[a] ages 20–74 by sex, England 1981–87

| *Job group* | *Title* | *Number of registrations* *Men* | *Women* | *Total* |
|---|---|---|---|---|
| 29 | Electrical and electronic engineers (professional) | 450 | 29 | 479 |
| 136 | Electrical and electronic production fitters | 172 | 6 | 178 |
| 137 | Electricians | 3141 | 8 | 3149 |
| 138 | Electrical plant operators | 516 | 9 | 525 |
| 139 | Telephone fitters | 1089 | 26 | 1115 |
| 140 | Cable jointers and linesmen | 233 | 5 | 238 |
| 141 | Radio and TV mechanics | 326 | 9 | 335 |
| 142 | Other electronic maintenance engineers | 259 | 9 | 268 |
| 143 | Electrical engineers (so described) | 1152 | 31 | 1183 |
| 155 | Electronics wiremen | 72 | 49 | 121 |
| 156 | Coil winders | 36 | 58 | 94 |
| 161 | Electrical, electronic assemblers | 85 | 211 | 296 |
| | Total | 7531 | 450 | 7981 |

[a]Electrical workers were defined using the Southampton Occupational Classsification (Coggon *et al*., 1995).

myeloid leukaemia alone (PRR = 128, 95% CI = 103–160, $P = 0.04$). Significantly reduced risks were observed for liver cancer (PRR = 69, 95% CI = 49–96, $P = 0.03$) and lung cancer (PRR = 86, 95% CI = 82–90, $P < 0.001$). The statistically significant results observed for both sexes combined remained evident when cancer registrations for men were analysed separately, among whom an increased risk of bladder cancer was also seen (PRR = 110, 95% CI = 101–120, $P = 0.04$). Among women, the only significant finding was an excess of malignant brain cancer (PRR = 202, 95% CI = 105–353, $P = 0.04$).

For cancer sites that were either significantly increased or decreased in analyses of both sexes combined or in males alone, PRRs for men were calculated separately for the age groups 20–64 years and 65–74 years (Table III). For several cancers, most notably malignant brain and acute myeloid leukaemia, the increased risks were most evident in men under 65 years. For other cancers, including cancers of the lung and pleura, PRRs were similar for both age groups. Examination of the PRRs for each of the 12 job groups listed in Table I did not reveal any unusual patterns, although the number of registrations in some groups was small (data not shown).

Of the 114 pleural cancers observed in male electrical workers, 88% had their histology coded as mesothelioma, a similar percentage being observed for pleural cancers among men in all other occupations (data not shown). Histological subtypes of lung cancer among male electrical workers were also similar to those of men in all other occupations (data not shown).

The age-adjusted proportion of current smokers among male electrical workers was significantly lower than that of men in all other occupations (PCSR = 85, 95% CI = 76–96, $P = 0.01$). Among men, PRRs for sites other than lung that have been related to tobacco exposure [oral cavity, oesphagus, pharynx, pancreas (Doll *et al*., 1994)] were equivocal, apart from laryngeal cancer, which showed a borderline deficit (PRR = 81, 95% CI = 65–100, $P = 0.06$) and bladder cancer, which was raised (PRR = 110, 95% CI = 101–120, $P = 0.04$).

### Discussion

The analyses presented here confirm previously published reports that electrical workers are at a significantly increased risk of pleural mesothelioma (Peto *et al*., 1995). Four cancers have previously been linked with exposure to ELF-EMF: leukaemia, brain, breast (in both men and women) and melanoma (Wiklund *et al*., 1981; Milham, 1982, 1985; Vägerö and Olin, 1983; Stern *et al*., 1986; Thomas *et al*., 1987; DeGuire *et al*., 1988; Pearce *et al*., 1989; Bastuji-Garin *et al*., 1990; Garland *et al*., 1990; Juutilainen *et al*., 1990; Loomis and Savitz, 1990; Tynes and Andersen, 1990; Demers *et al*., 1991; Floderus *et al*., 1993; Guénel *et al*., 1993; London *et al*., 1994; Loomis *et al*., 1994; Thériault *et al*., 1994; Savitz and Loomis, 1995). Support is provided here for two of these cancers: leukaemia and brain, where significant excesses of around 20% were observed. Although excesses were also noted for melanoma and male breast cancer, the numbers of events for these cancers were smaller and the risks were not significantly raised. The 10% deficit observed for female breast cancer was likewise not statistically significant and offers little support for the suggestion that this malignancy is affected by exposure to ELF-EMF (Loomis *et al*., 1994). The low PRR observed for lung cancer may reflect less smoking among electrical workers compared with other occupational groups. This interpretation is given some support by the proportional current smoking ratio (PCSR), which suggests that current smoking among electrical workers in the 1980s was on average 15% lower than that of other occupational groups. Unfortunately, information on past smoking habits was not available.

The findings described in this paper have the advantage of being based on an analysis of a very large population-based data set obtained from routine cancer registrations. The interpretation of these data are not straightforward, however, and several issues need to be considered. These have been discussed in detail elsewhere (Elliott, 1995; Roman and Carpenter, 1995). Briefly, they include the lack of appropriate denominator data, the need to exclude a large percentage of individuals with inadequately described occupations (48% in men, 71% in women), the reliance on occupational titles recorded at the time of cancer registration, the limited amount of information on confounding factors and lack of data on specific occupational exposures. Furthermore, in analyses in which many associations are examined, some results may be significantly high or low by chance alone. Accordingly, statistically significant associations arising from these analyses that have not been reported previously should be interpreted with caution.

The lack of appropriate denominator data deserves particular attention since the PRRs for relatively rare cancers, such as pleura, may be disproportionately affected by the low or high incidence of common cancers, such as lung. In order to address this issue, we have applied an approach suggested by McDowall (1983). This involved recalculating PRRs for cancers of the pleura, brain and leukaemia after a stepwise exclusion from the analysis of the four most common cancers registered in men and women with an adequately described occupation [men: cancers of the lung, skin other than melanoma, stomach and bladder; women: cancers of the breast, *in situ* cervix, lung and skin other than melanoma (Roman and Carpenter, 1995)] (Table IV). While the exclusion of the most common cancer (lung cancer for men and breast cancer for women) resulted in a reduction of the PRRs for each of the three cancers examined, the general constancy of the PRRs across the analyses suggests that the main results for these cancers were not seriously biased.

Further support for our findings comes from the previous Decennial Supplement on occupational mortality, which analysed deaths registered in the years 1979–80 and 1982–83 using denominators from the 1981 Census to estimate standardised mortality ratios (SMRs) (OPCS, 1986). SMRs for pleural cancer, brain cancer and leukaemia among electrical workers were all significantly raised (pleural cancer SMR = 229, 95% CI = 159–330, based on 29 deaths; brain cancer SMR = 121, 95% CI = 101–145, based on 119 deaths; leukaemia SMR = 142, 95% CI = 117–172, based on 106 deaths).

The possible aetiological role of exposure to ELF-EMF in the associations noted here, and elsewhere, is not clear. The fact that a number of studies of electrical workers have reported raised risks of leukaemia and brain cancer is noteworthy. At present, however, the epidemiological evidence for a carcinogenic effect of ELF-EMF is limited. While theories abound, a biologically plausible mechanism whereby the carcinogenic processes could be influenced by exposure to ELF-EMF has yet to be established (Doll, 1994).

**Table II** Adjusted proportional registration ratios (PRR)[a] and 95% confidence intervals (CI) for electrical workers[b] aged 20–74; England, 1981–87

| | *Men* | | *Women* | | *Total* | |
|---|---|---|---|---|---|---|
| *Cancer (IDC code)* | *Number observed* | *Adjusted PRR[a] (95% CI)* | *Number observed* | *Adjusted PRR[a] (95% CI)* | *Number observed* | *Adjusted PRR[a] (95% CI)* |
| Oral cavity (141, 143–145) | 56 | 93 (71–122) | 2 | 109 (13–396) | 58 | 94 (73-122) |
| Salivary (142) | 8 | 70 (31–140) | 0[c] | – | 8 | 67 (29–132) |
| Pharynx (146–148) | 45 | 90 (66–121) | 2 | 181 (22–655) | 47 | 92 (69–123) |
| Oesophagus (150) | 206 | 113 (99–130) | 5 | 95 (31–224) | 211 | 113 (98–129) |
| Stomach (151) | 513 | 99 (91–108) | 14 | 100 (55–169) | 527 | 99 (91–108) |
| Small intestine (152) | 17 | 127 (74–204) | 0[c] | – | 17 | 124 (77–200) |
| Colon (153) | 413 | 107 (97–118) | 15 | 72 (41–120) | 428 | 105 (96–116) |
| Rectum (154) | 360 | 105 (95–117) | 15 | 128 (72–212) | 375 | 106 (96–118) |
| Liver (155) | 33 | 68 (48–97)* | 1 | 63 (2–352) | 34 | 69 (49–96)* |
| Gallbladder (156) | 32 | 85 (58–120) | 4 | 160 (44–411) | 36 | 90 (65–124) |
| Pancreas (157) | 236 | 102 (90–116) | 4 | 45 (12–116) | 240 | 102 (90–116) |
| Retroperitoneum (158.0) | 8 | 120 (52–238) | 0[c] | – | 8 | 118 (51-232) |
| Peritoneum (158.8–158.9) | 7 | 149 (60–308) | 0[c] | – | 7 | 146 (70–306) |
| Nose and nasal sinuses (160) | 11 | 65 (33–118) | 0[c] | – | 11 | 64 (35–115) |
| Larynx (161) | 88 | 81 (65–100) | 3 | 203 (42–595) | 91 | 83 (67–102) |
| Lung (162) | 1935 | 85 (81–89)*** | 58 | 111 (85–144) | 1993 | 86 (82–90)*** |
| Pleura (163) | 114 | 201 (166–242)*** | 1 | 164 (4–915) | 115 | 201 (167–241)*** |
| Thymus and mediastinum (164) | 11 | 114 (57–205) | 1 | 451 (12–2518) | 12 | 122 (70–216) |
| Bone (170) | 22 | 120 (76–183) | 2 | 349 (42–1262) | 24 | 128 (86–190) |
| Soft tissue (171) | 36 | 103 (73–143) | 1 | 67 (2–374) | 37 | 102 (74–141) |
| Melanoma (172) | 83 | 118 (94–147) | 8 | 127 (55–251) | 91 | 119 (97–146) |
| Skin other than melanoma (173) | 624 | 109 (102–119)* | 29 | 109 (74–158) | 653 | 110 (102–119)* |
| Female breast (174) | – | – | 83 | 89 (72–112) | 83 | 89 (72–112) |
| Male breast (175) | 14 | 129 (71–217) | – | – | 14 | 129 (71–217) |
| Uterus (179, 181, 182) | – | – | 11 | 83 (42–149) | 11 | 83 (42–149) |
| Cervix (180) | – | – | 24 | 109 (70–163) | 24 | 109 (70–163) |
| *In situ* cervix (233.1) | – | – | 58 | 97 (74–126) | 58 | 97 (74–126) |
| Ovary (183) | – | – | 22 | 106 (67–161) | 22 | 106 (67–161) |
| Prostate (185) | 381 | 102 (93–113) | – | – | 381 | 102 (93–113) |
| Testis (186) | 139 | 104 (88–123) | – | – | 139 | 104 (88–123) |
| Other male genital organs (187) | 25 | 122 (79–180) | – | – | 25 | 122 (79–180) |
| Bladder (188, 189.1–189.9) | 476 | 110 (101–120)* | 5 | 58 (19–137) | 481 | 109 (100–119) |
| Kidney (except pelvis) (189.0) | 147 | 108 (92–127) | 2 | 44 (5–161) | 149 | 106 (91–125) |
| Eye (190) | 12 | 98 (51–172) | 1 | 158 (4–882) | 13 | 102 (59–175) |
| Brain and meninges (191, 192, 225, 237.5–237.9, 239.6) | 267 | 114 (102–129)* | 14 | 140 (77–236) | 281 | 116 (103–130)* |
| Malignant brain (191) | 192 | 115 (100–133) | 12 | 202 (105–353)* | 204 | 118 (103–136)* |
| Thyroid (193) | 21 | 106 (66–163) | 2 | 80 (10–291) | 23 | 104 (69–156) |
| Suprarenal and other endocrine organs (194) | 8 | 139 (60–275) | 0[c] | – | 8 | 131 (57–258) |
| Ill-defined and secondary (195–199) | 370 | 95 (86–106) | 15 | 88 (50–146) | 385 | 95 (86–105) |
| Non-Hodgkin's lymphoma (200, 202) | 221 | 108 (95–124) | 7 | 89 (36–183) | 228 | 108 (94–122) |
| Hodgkin's disease (201) | 97 | 111 (91–136) | 4 | 155 (42–397) | 101 | 113 (93–137) |
| Myeloma (203) | 101 | 136 (111–166)** | 2 | 72 (9–264) | 103 | 134 (111–163)** |
| All leukaemias (204–208) | 208 | 123 (108–142)** | 9 | 143 (66–272) | 217 | 124 (109–142)** |
| Acute lymphatic leukaemia (204.0) | 17 | 122 (71–196) | 0[c] | – | 17 | 116 (72–186) |
| Chronic lymphatic leukaemia (204.1) | 48 | 114 (84–151) | 1 | 97 (3–541) | 49 | 114 (86–151) |
| Acute myeloid leukaemia (205.0) | 77 | 129 (102–162)* | 3 | 101 (21–296) | 80 | 128 (103–160)* |
| Chronic myeloid leukaemia (205.1) | 30 | 108 (73–154) | 2 | 217 (27–787) | 32 | 112 (79–158) |

[a]PRRs are adjusted for age (5 year age groups), social class (six classes), cancer registry (13 registries) and sex (where appropriate). [b]Electrical workers were defined using the Southampton Occupational Classification (including the following job groups: 29, 136, 137, 138, 139, 140, 141, 142, 143, 155, 156, 161) (Coggon *et al.*, 1995). [c]No registrations observed and $P > 0.05$. *$P < 0.05$ **$P < 0.01$. ***$P < 0.001$.

**Table III** Adjusted proportional registration ratios (PRR)[a] and 95% confidence intervals (CI) for cancers of the liver, lung, pleura, other skin, bladder, brain and meninges, myeloma and leukaemia among men employed as electrical workers[b] by age, England 1981–87

| Cancer (ICD Code) | Ages 20–64 Number observed | Ages 20–64 Adjusted PRR[a] (95% CI) | | Ages 60–74 Number observed | Ages 60–74 Adjusted PRR[a] (95% CI) | | Ages 20–74 Number observed | Ages 20–74 Adjusted PRR[a] (95% CI) | |
|---|---|---|---|---|---|---|---|---|---|
| Liver (155) | 11 | 39 | (20–71)*** | 22 | 110 | (69–167) | 33 | 68 | (48–97)* |
| Lung (162) | 974 | 82 | (78–88)*** | 961 | 87 | (82–94)*** | 1935 | 85 | (81–89)*** |
| Pleura (163) | 78 | 196 | (155–245)*** | 36 | 211 | (149–294)*** | 114 | 201 | (166–242)*** |
| Other skin (173) | 404 | 106 | (96–117) | 220 | 117 | (103–134)* | 624 | 109 | (102–119)* |
| Bladder (188, 189.1–189.9) | 270 | 104 | (93–118) | 206 | 117 | (102–134)* | 476 | 110 | (101–120)* |
| Brain and meninges (191, 192, 225, 237.5–237.9, 239.6) | 211 | 114 | (100–131) | 56 | 117 | (89–152) | 267 | 114 | (102–129)* |
| Malignant brain (191) | 158 | 118 | (101–138)* | 34 | 103 | (72–145) | 192 | 115 | (100–133) |
| Myeloma (203) | 64 | 146 | (113–187)** | 37 | 122 | (86–169) | 101 | 136 | (111–166)** |
| All leukaemias (204–208) | 143 | 134 | (113–158)*** | 65 | 105 | (82–135) | 208 | 123 | (108–142)** |
| Acute myeloid leukaemia (205.0) | 58 | 147 | (112–190)** | 19 | 94 | (57–148) | 77 | 129 | (102–162)* |

[a]PRRs are adjusted for age (5 year age groups), social class (six classes) and cancer registry (13 registries). [b]Electrical workers were defined using the Southampton Occupational Classification. (including the following job groups: 29, 136, 137, 138, 139, 140, 141, 142, 143, 155, 156, 161) (Coggon *et al.*, 1995). *$P<0.05$. **$P<0.01$. ***$P<0.001$.

**Table IV** Adjusted proportional registration ratios (PRR)[a] and 95% confidence intervals (CI) for cancers of the pleura, brain and leukaemia for electrical workers[b] aged 20–74 in England, 1981–87, showing the effect of stepwise removal from the analysis of the four most common cancers registered in all men and women with an adequately described occupation

| | Adjusted PRR[a] (95% CI) Pleural cancer (ICD 163) | | Malignant brain cancer (ICD 191) | | All leukaemias (ICD 204–208) | |
|---|---|---|---|---|---|---|
| Original PRR | 201 | (167–241) | 118 | (103–136) | 124 | (109–142) |
| PRR based on all causes less the most common cancer[c] | 187 | (156–224) | 112 | (98–128) | 118 | (103–135) |
| PRR based on all causes less the two most common cancers[d] | 186 | (155–223) | 112 | (98–129) | 118 | (104–135) |
| PRR based on all causes less the three most common cancers[e] | 184 | (154–221) | 112 | (97–128) | 117 | (102–134) |
| PRR based on all causes less the four most common cancers[f] | 184 | (153–221) | 111 | (97–128) | 117 | (103–134) |

[a]PRRs are adjusted for age (5 year age groups), social class (6 classes), cancer registry (13 registries) and sex. [b]Electrical workers were defined using the Southampton Occupational Classification (including the following job groups: 29, 136, 137, 138, 139, 140, 141, 142, 143, 155, 156, 161) (Coggon *et al.*, 1995). [c]Men, lung cancer; women, breast cancer. [d]Men, cancers of the lung and skin other than melanoma, women, cancers of the breast and *in situ* cervix. [e]Men, cancers of the lung, skin other than melanoma and stomach; women, cancers of the breast, *in situ* cervix and lung. [f]Men, cancers of the lung, skin other than melanoma, stomach and bladder; women, cancers of the breast, *in situ* cervix, lung and skin other than melanoma.

In addition, most of the epidemiological studies of the putative health effects of ELF-EMF have used a surrogate marker of exposure (such as occupational title, as in this study) rather than direct measurement. The possibility that the observed associations are due to workplace exposures other than ELF-EMF, such as chemicals, should not be discounted.

The majority of pleural cancers among male electrical workers in this study were coded as mesothelioma, and the observed doubling of pleural cancer risk underlines the seriousness of this occupational exposure. In contrast to the uncertainty surrounding the carcinogenic effects of ELF-EMF, the excess of mesothelioma among electrical workers has a strong foundation and is likely to result from asbestos exposure (Enterline *et al.*, 1987; Peto *et al.*, 1995). Asbestos exposure has also been associated with peritoneal cancer (Peto *et al.*, 1995). A 50% excess of peritoneal cancer was observed in these data but this was based on only seven cases and was not statistically significant.

In conclusion, these findings confirm the previously observed association between pleural cancer and exposure to asbestos in electrical workers. The extent to which workplace exposures to ELF-EMF explains the excesses seen here for leukaemia and brain cancer requires further study.

**Acknowledgements**
We would like to thank Jane Roberts for help with data management and statistical analyses, and Valerie Beral and Richard Doll for their helpful comments on the manuscript. We thank the Office of Population, Censuses and Surveys for supplying the cancer registry data for the purpose of epidemiological analysis. RN is supported by an MRC research training fellowship.

### References

BASTUJI-GARIN S, RICHARDSON S AND ZITTOUN R. (1990). Acute leukaemia in workers exposed to electromagnetic fields. *Eur. J. Cancer*, **26**, 1119–1120.

BRESLOW NE AND DAY NE. (1987). *Statistical Methods in Cancer Research. Vol. II. The Design and Analysis of Cohort Studies.* IARC Scientific Publications No. 82. IARC: Lyon.

COGGON D, INSKIP H, WINTER P AND PANNETT B. (1995). Appendix 2: Definition of the Southampton classification of job groups. In *Occupational Health. Decennial Supplement for England and Wales*, Registrar General. pp. 282–291. HMSO: London.

DEGUIRE L, THÉRIAULT G, ITURRA H, PROVENCHER S, CYR D AND CASE BW. (1988). Increased incidence of malignant melanoma of the skin in workers in a telecommunications industry. *Br. J. Ind. Med.*, **45**, 824–828.

DEMERS PA, THOMAS DB, ROSENBLATT KA, JIMENEZ LM, MCTIREN A, STALSBERG H, STERNHAGEN A, THOMPSON WD, CURNEN MG, SATARIANO W, AUSTIN DF, ISACSON P, GREENBERG RS, KEY C, KOLONEL LN AND WEST DE. (1991). Occupational exposure to electromagnetic fields and breast cancer in men. *Am. J. Epidemiol.*, **134**, 340–347.

DOLL R. (1994). Electromagnetic fields and the risk of cancer. Supplementary Report by the Advisory Group on Non-Ionising Radiation. In *Documents of the NRPB. Health Effects related to the use of Visual Display Units*. Vol. 5. No. 2, National Radiological Protection Board. pp. 78–81. HMSO: London.

DOLL R, PETO R, WHEATLEY K, GRAY R AND SUTHERLAND I. (1994). Mortality in relation to smoking: 40 years observations on male British doctors. *Br. Med. J.*, **309**, 901–911.

ELLIOTT R. (1995). Chapter 12: Smoking, drinking and occupation. In *Occupational Health. Decennial Supplement for England and Wales*, Registrar General. pp. 195–216. HMSO: London.

ENTERLINE PE, HARTLEY J AND HENDERSON V. (1987). Asbestos and cancer: a cohort followed up to death. *Br. J. Ind. Med.*, **44**, 396–401.

FLODERUS B, PERSSON T, STENLUND C, WENNBERG A, OST A AND KNAVE B. (1993). Occupational exposure to electromagnetic fields in relation to leukaemia and brain tumours: a case–control study in Sweden. *Cancer Causes Control*, **4**, 465–476.

GARLAND FC, SHAW E, GORHAM ED, GARLAND CF, WHITE MR AND SINSHEIMER PJ. (1990). Incidence of leukaemia in occupations with potential electromagnetic field exposure in United States Navy personnel. *Am. J. Epidemiol.*, **132**, 293–303.

GUÉNEL P, RASKMARK P, ANDERSEN JB AND LYNGE E. (1993). Incidence of cancer in persons with occupational exposure to electromagnetic fields in Denmark. *Br. J. Ind. Med.*, **50**, 758–764.

JUUTILAINEN J, LAARA E AND PUKKALA E. (1990). Incidence of leukaemia and brain tumours in Finnish workers exposed to ELF magnetic fields. *Int. Arch. Occup. Environ. Health*, **62**, 289–293.

LONDON SJ, BOWMAN JD, SOBEL E, THOMAS DC, GARABRANT DH, PEARCE N, BERNSTEIN L AND PETERS JM. (1994). Exposure to magnetic fields among electrical workers in relation to leukaemia risk in Los Angeles county. *Am. J. Ind. Med.*, **26**, 47–60.

LOOMIS DP AND SAVITZ DA. (1990). Mortality from brain cancer and leukaemia among electrical workers. *Br. J. Ind. Med.*, **47**, 633–638.

LOOMIS DP, SAVITZ DA AND ANANTH CV. (1994). Breast cancer mortality among female electrical workers in the United States. *J. Natl Cancer Inst.*, **86**, 921–925.

MCDOWALL M. (1983). Adjusting proportional mortality ratios for the influence of extraneous causes of death. *Stat. Med.*, **2**, 467–475.

MILHAM S. (1982). Mortality from leukaemia in workers exposed to electrical and magnetic fields. *N. Engl. J. Med.*, **307**, 249.

MILHAM S. (1985). Mortality in workers exposed to electromagnetic fields. *Environ. Health Perspect.*, **62**, 297–300.

NATIONAL RADIOLOGICAL PROTECTION BOARD. (1992). *Documents of the NRPB. Electromagnetic Fields and the Risk of Cancer*. Vol. 3. No. 1. HMSO: London.

OFFICE OF POPULATION, CENSUSES AND SURVEYS. (1980). *Classification of Occupations 1980*. HMSO: London.

OFFICE OF POPULATION, CENSUSES AND SURVEYS. (1986). *Occupational Mortality 1979–80, 1982–8. Decennial Supplement for Great Britain*. HMSO: London.

OFFICE OF POPULATION, CENSUSES AND SURVEYS. (1988). *General Household Survey 1986*. HMSO: London.

OFFICE OF POPULATION, CENSUSES AND SURVEYS. (1990). *General Household Survey 1988*. HMSO: London.

OFFICE OF POPULATION, CENSUSES AND SURVEYS. (1992). *General Household Survey 1990*. HMSO: London.

PEARCE NE, REIF J AND FRASER J. (1989). Case–control studies in New Zealand electrical workers. *Int. J. Epidemiol.*, **18**, 55–59.

PETO J, HODGSON JT, MATTHEWS FE AND JONES JR. (1995). Continuing increase in mesothelioma mortality in Britain. *Lancet*, **345**, 535–539.

ROMAN E AND CARPENTER L. (1995). Chapter 7: Cancer Incidence in England 1981–1987. In *Occupational Health. Decennial Supplement for England and Wales*, Registrar General. pp. 77–102. HMSO: London.

SAVITZ DA AND LOOMIS DP. (1995). Magnetic field exposure in relation to leukaemia and brain cancer mortality among electric utility workers. *Am. J. Epidemiol.*, **141**, 123–134.

STERN FB, WAXWEILER RA, BEAUMONT JJ, LEE ST, RINSKY RA, ZUMWALDE RD, HALPERIN WE, BIERBAUM PJ, LANDRIGAN PJ AND MURRAY WE (1986). A case–control study of leukaemia at a naval nuclear shipyard. *Am. J. Epidemiol.*, **123**, 980–992.

THÉRIAULT G, GOLDBERG M, MILLER AB, ARMSTRONG B, GUÉNEL P, DEADMAN J, IMBERNON E, TO T, CHEVALIER A, CYR D AND WALL C. (1994). Cancer risks associated with occupational exposure to magnetic fields among electric utility workers in Ontario and Quebec, Canada and France: 1970–1989. *Am. J. Epidemiol.*, **139**, 550–572.

THOMAS TL, STOLLEY PD, STERNHAGEN A, FONTHAM ET, BLEECKER ML, STEWART PA AND HOOVER RN. (1987). Brain tumour mortality risk among men with electrical and electronics jobs: a case-control study. *J. Natl Cancer Inst.*, **79**, 233–238.

TYNES T AND ANDERSEN A. (1990). Electromagnetic fields and male breast cancer. *Lancet*, **336**, 1596.

VÄGERÖ D AND OLIN R. (1983). Incidence of cancer in the electronics industry: using the new Swedish cancer environment registry as a screening instrument. *Br. J. Ind. Med.*, **40**, 188–192.

WIKLUND K, EINHORN J AND EKLUND G. (1981). An application of the Swedish cancer-environment registry. Leukaemia among telephone operators at the telecommunications administration in Sweden. *Int. J. Epidemiol.*, **10**, 373–376.

WORLD HEALTH ORGANIZATION. (1977). *International Classification of Diseases*. Ninth ed. Manual of the International Statistical Classification of Diseases, Injuries and Causes of Death. Vol. 1. WHO: Geneva.